# Bioactive Absorbent Chitosan Aerogels Reinforced with Bay Tree Pruning Waste Nanocellulose with Antioxidant Properties for Burger Meat Preservation

**DOI:** 10.3390/polym15040866

**Published:** 2023-02-09

**Authors:** Esther Rincón, Eduardo Espinosa, María Pinillos, Luis Serrano

**Affiliations:** BioPren Group (RNM-940), Chemical Engineering Department, Instituto Químico para la Energía y el Medioambiente (IQUEMA), Faculty of Science, Universidad de Córdoba, 14014 Cordoba, Spain

**Keywords:** bay tree, food packaging pads, bioactive aerogels, residual lignin, meat preservation

## Abstract

As a transition strategy towards sustainability, food packaging plays a crucial role in the current era. This, carried out in a biorefinery context of agricultural residues, involves not only obtaining desirable products but a comprehensive utilization of biomass that contributes to the circular bioeconomy. The present work proposes the preparation of bioactive absorbent food pads through a multi-product biorefinery approach from bay tree pruning waste (BTPW). In a first step, chitosan aerogels reinforced with lignocellulose and cellulose micro/nanofibers from BTPW were prepared, studying the effect of residual lignin on the material’s properties. The presence of micro/nanofibers improved the mechanical performance (up to 60%) in addition to increasing the water uptake (42%) when lignin was present. The second step was to make them bioactive by incorporating bay leaf extract. The residual lignin in the micro/nanofibers was decisive, since when present, the release profiles of the extract were faster, reaching an antioxidant power of more than 85% after only 30 min. Finally, these bioactive aerogels were used as absorbent pads for fresh meat. With the use of the bioactive aerogels (with ≥2% extract), the meat remained fresh for 10 days as a result of delayed oxidation of the food during storage (20% metmyoglobin proportion).

## 1. Introduction

Food losses and food waste are some of the biggest concerns for modern society and the food industry. In addition, foodborne outbreaks pose a threat to public health. These facts make the market for the food industry increasingly demanding, and in this context, packaging plays a crucial role. This is because packaging can not only protect the food from the external environment and maintain its quality but also add functionalities that improve the nutritional value of the food product by incorporating compounds that are beneficial to health, such as antioxidant compounds [1,2].

In the specific case of meat packaging, absorbent pads are commonly used for moisture control as they absorb the excess liquid released by meat during storage. When it is desired to apply absorbent pads in meat preservation, the ideal would be materials with antioxidant capacity to inhibit the oxidation process that occurs during meat storage and compromises its quality. Thus, the presence of antioxidant compounds is expected to reduce the oxidation of polyunsaturated acids [3,4]. The architecture of these pads is usually two-layered: an upper layer made from impermeable and non-adherent synthetic polymers, such as polyethylene, and a hydrophilic non-woven lower layer containing active substances that inhibit bacterial growth [5]. However, due to the serious environmental problems associated with the manufacture of synthetic plastics and additives, food packaging industries are looking for new alternatives that prioritize the use of more sustainable bio-based and renewable materials, such as biopolymers and natural extracts [1]. Lignocellulosic biomass is an ideal source to obtain biopolymers and antioxidant natural compounds. In fact, biopolymers and polyphenolic compounds isolated from different plant biomasses have already demonstrated their potential for use in the food packaging industry [6,7,8,9,10]. Cellulose and nanocellulose from different sources (vine shoot, wheat straw, or eucalyptus sawdust, among others) have been used for the preparation of biocomposite films [11,12,13]. Lignin and hemicelluloses have been successfully applied as natural additives in food packaging materials [14,15,16]. In this context, research has also been conducted in recent years on the preparation of absorbent pads from natural biopolymers for food packaging [1,17]. Fonseca et al. prepared aerogels from corn starch with pinhão coat extract that showed a high capacity for water absorption and as carriers of phenolic compounds [18]. Along the same lines, de Oliveira et al. prepared bioactive aerogels with desirable properties for food packaging from cellulose and nanocellulose isolated from yerba-mate [19].

Among natural polymers, chitosan has received special attention in recent years due to its excellent biocompatibility and biodegradability, as well as its non-toxic and antimicrobial properties [20]. On the other hand, aerogels have also emerged in recent years as relevant materials due to the characteristics they offer. Aerogels are materials obtained from a gel from which the fluid is removed through its pores, resulting in very specific physical properties such as low density, very high porosity, and high specific surface area [21]. However, chitosan aerogels have some shortcomings such as low porosity, irregular structure, and easy deformation, thus limiting their applications [22,23]. These drawbacks could be overcome by reinforcing the chitosan aerogel with another biopolymer having the desired characteristics. Cellulose nanofibers are a very attractive candidate for the preparation of aerogels due to their physical and chemical properties (high strength, adaptable pores, and modifiable surface properties), in addition to their abundant availability [24]. As previously mentioned, cellulose is the main biopolymer present in lignocellulosic biomass and, therefore, in agricultural residues that need to be revalued to achieve a circular economy. Besides this, numerous studies have reported the importance of the presence or absence of residual lignin in fibers for various applications from fiberboard production to the preparation of PLA or PVA biocomposites [12,25,26]. The presence of residual lignin presents some beneficial advantages for the food packaging industry, such as lower hydrophilicity, high dispersion and reinforcing capacity, and lower energy and economic requirements in its production since no additional pulp bleaching process is needed [27,28]. On the other hand, the presence of lignin increases the roughness and gives a dark color to the fibers but also increases the antioxidant power due to the aromatic structure of lignin [12]. The use of lignin-free fibers results in a brighter, cleaner pulp, free of shavings and bark. The lignin-free fibers can swell more freely, increasing their flexibility and malleability [26]. However, no studies have reported the effect of the presence of residual lignin in nanocellulose for absorbing food pads.

In this context, the idea of using BTPW to obtain polyphenolic compounds, which serve as bioactive compounds, and nanocellulose, which serves as a reinforcing agent and structure and performance improver, through multi-product biorefinery processes for the formulation of chitosan-based aerogels is explored. The bay tree (*Laurus nobilis*) is an abundant softwood in the Mediterranean area whose purposes, in addition to the culinary, have been oriented to the application of its essential oils in cosmetics or medicine, leaving unused a large amount of residue. Previously, the valorization of this biomass has been addressed in terms of its natural extracts and hemicelluloses [15,29,30]. However, as far as the authors know, the purification of cellulose from bay tree pruning waste has not been addressed.

Thus, in the first stage, the present work deals with the obtention of cellulose micro/nanofibers from bay tree pruning waste, conducting a proper characterization and evaluation of their use as a reinforcing agent for chitosan aerogels, considering the effect of residual lignin in the fibers. Subsequently, these aerogels have been functionalized with bay leaf extract, whose beneficial properties in food packaging materials have been previously reported [31]. Lastly, the antioxidant capacity of the bioactive aerogels was tested together with their application as absorbing pads for meat preservation in burger samples. As previously discussed, the presence or absence of residual lignin in cellulose nanofibers results in usable properties for food packaging composites. So far, no studies have been conducted on the effect of this residual lignin in bioactive absorbent food pads. The use of lignocellulose micro/nanofibers (LCMNFs) from unbleached cellulose pulp applied in these materials could lead to an increase in water absorption capacity and antioxidant power due to the presence of lignin, in addition to being a more economical and environmentally friendly process that does not require a bleaching step. On the other hand, the use of cellulose micro/nanofibers (CMNFs) obtained from bleached cellulose pulp could result in more flexible materials that are easier to functionalize with bioactive compounds. For these reasons, in the present work, it was decided to simultaneously study BTPW-LCMNFs and CMNFs.

## 2. Materials and Methods

### 2.1. Materials

Bay tree pruning waste (BTPW) was kindly supplied by an independent farmer from Arjonilla (37°58′27″ N-4°06′27″ W) in the province of Jaén, Spain.

The following reagents were used during the pulping process and cellulose micro/nanofibers production and characterization: sodium hydroxide (NaOH, pellets, pure, pharma grade, CAS: 1310-73-2), hydrochloric acid (HCl, 37%, CAS: 7647-01-0), acetic acid glacial (99.7% purity, CAS: 64-19-7), and Copper(II) Ethylenediamine (CAS: 14552-35-3) from PanReac AppliChem, ITW Reagents; sodium chlorite (NaClO, 80% purity, CAS: 7758-19-2) and sodium bromide (NaBr, 99%, CAS: 7647-15-6) from Honeywell, Fluka^TM^; 2,2,6,6,-Tetramethyl-1-piperidinyloxy (TEMPO, free radical, 98% purity, CAS: 2564-83-2) from Sigma-Aldrich; Poly-Dacmac (0.001 N, CAS: 26062-79-3) and Pes-Na (0.001 N, CAS: 25704-18-1) from BTG. 

The reagents used during aerogel preparation and characterization were chitosan (high molecular weight 310,000–375,000 Da, >75% deacetylated chitin, Poly (D-glucosamine), CAS: 9012-76-4) purchased from Sigma-Aldrich, ethanol (96.0%, PanReac AppliChem ITW Reagents, CAS: 64-17-5), 2,2-Diphenyl-1-picrylhydrazyl (DPPH, Sigma-Aldrich, CAS: 1898-66-4), soybean oil (CAS: 8001-22-7) from Guinama S.L.U., potassium dihydrogen phosphate (CAS: 7778-77-0) and di-Potassium hydrogen phosphate (CAS: 7758-11-4) from LabKem labbox.

### 2.2. Production and Characterization of Bay Leaf Extract

Bay leaf extract (BT) was obtained using the Soxhlet method. For this, 5 g of biomass (dry weight basis) were placed into the Soxhlet extraction thimble and placed in the extraction equipment. The extraction was performed using 200 mL of pure ethanol for 5 h (until no solvent coloration was observed). Once the extraction was finished, BT was cooled to room temperature and filtered through a filter paper cone (Whatman nº1).

The total phenolic content (TPC) of the extract was determined following the Folin–Ciocalteu method as follows. Folin–Ciocalteu reagent (1.25 mL) was mixed with 2.5 mL of aqueous sodium carbonate (saturated solution), 0.25 mL of BT, and made up to a final volume of 25 mL with distilled water. The sample was incubated for 30 min at 40 °C in a thermostatic bath, and then its absorbance was read at 760 nm. The TPC was calculated from a calibration curve of gallic acid (100–1000 mg/L gallic acid) and expressed as mg gallic acid equivalents per gram of BT (GAE/g).

The radical scavenging activity of BT was determined using the DPPH method. Briefly, a 0.2 mM DPPH solution in methanol was prepared, and its absorbance was read at 517 nm. Then, 980 µL of DPPH methanolic solution was mixed with 20 µL of BT and incubated for 30 min in the dark before measuring the absorbance at 517 nm. The DPPH radical scavenging activity (%SA) was calculated according to the Equation (1):

(1) $$SA(\%)=\frac{ABS_{blank}-ABS_{sample}}{ABS_{blank}}\times 100$$

### 2.3. BTPW Cellulose Pulp Production

BTPW was pulped in a 15 L batch reactor at 120 ± 1 °C for 90 min containing 12% NaOH (o.d.m.) solution, heated by an outer jacket heater and stirred by rotating the reaction vessel via a motor; the liquid/solid ratio was 8:1. After pulping, cooked material was dispersed in a pulp disintegrator at 1200 rpm for 30 min. The pulp was then passed through a Sprout-Bauer beater and separated by sieving through a netting of 0.14 mm mesh size, obtaining the unbleached semichemical pulp (UBP). Bleached pulp (BP) was obtained by a bleaching process using 3 g of sodium chlorite per g of cellulose pulp in a 0.3% fiber suspension in water at 80 °C for 3 h. Once cooled, the pulp was filtered and washed with acetone and distilled water.

### 2.4. BTPW-LCMNF and CMNF Production

LCMNFs and CMNFs were obtained from the UBP and BP, respectively, being chemically pretreated. The chemical process consisted of a TEMPO-mediated oxidation [20]. A fiber suspension of 0.02% containing TEMPO (0.16 g) and NaBr (1 g) was prepared. The oxidation started adding 5 mmol of 12% NaClO solution with continuous stirring at room temperature. The pH was maintained at 10 by adding 0.5 M NaOH until no lowering pH was observed. Once the reaction finished, the oxidized fibers were washed and prepared at 1% aqueous suspension to pass through a high-pressure homogenizer (PANDA GEA 2 K NIRO) for 4 passes at 300 bar, 3 passes at 600 bar, and 3 passes at 900 bar.

### 2.5. BTPW Cellulose Fractions Characterization

#### 2.5.1. Characterization of BTPW Cellulose Pulps

BTPW cellulose pulps were characterized according to the standard protocols of the Technical Association of the Pulp and Paper Industry (TAPPI) to determine ashes (TAPPI T244), hot water solubility (TAPPI T1w-75), ethanol extractives (TAPPI T204), α-cellulose (TAPPI T203), hemicelluloses (TAPPI T212), lignin (TAPPI T222), viscosity (*η_s_*, TAPPI T-300m90) [32], and degree of polymerization (DP) [33].

#### 2.5.2. Characterization of LCMNF and CMNF

LCMNFs and CMNFs were characterized in terms of nanofibrillation yield, cationic demand, and carboxyl content.

The nanofibrillation yield (η) was determined gravimetrically after centrifugating in an Avanti J-25 centrifuge (Beckman, Brea, CA, USA) at 10,000 rpm for 12 min a 0.1% micro/nanofiber suspension to achieve the separation of the nanofibrillar material and non-nanofibrillated material.

The carboxyl content (CC) was measured by conductometric titration following the method of Besbes et al., 2011 [34].

The cationic demand (CD) was determined following the methodology of Espinosa et al., 2017 [35] using a Mütek PCD 05 particle charge detector. The specific surface area and theoretical diameter were calculated considering the stoichiometric relationship of the poly-Dadmac absorption on the hydroxyl and carboxyl groups of the micro/nanofibers surface.

Viscosity (*η_s_*) was measured according to TAPPI standard T-300m90 [32], and the data obtained were used to calculate the *DP* of the micro/nanofibers according to Equation (2) [36]

(2) $$DP=\frac{\eta_{S}}{0.42}$$

From the *DP* values, the length of the micro/nanofibers was calculated following the Equation (3) [37]

(3) $$Length\left(nm\right)=4.286\cdot DP-757$$

Lastly, all the BTPW cellulose fractions were examined using Fourier transform infrared spectroscopy (FTIR), X-ray diffraction, and scanning electron microscopy (SEM). FTIR analyses were performed using a spectrometer FTIR-ATR Perkin-Elmer Spectrum Two (Perkin Elmer, Waltham, MA, USA), in the range of 400–4000 cm^−1^ with a resolution of 4 cm^−1^ and a total of 40 scans. X-ray diffraction (XRD) analyses were performed using a Bruker D8 Discover (Bruker, Billerica, MA, USA) with a monochromatic source CuKα1 over an angular range of 10–80° at a scan speed of 0.025°/s in reflection mode. The background treatment was performed with lines connecting the minima. Samples were freeze-dried and compressed into flat sheets with a thickness of around 1 mm before measurement. The microscope was a JEOL JSM 7800F (Jeol, Tokyo, Japan). The acceleration voltage and working distance were 5 kV and 10 mm, respectively.

### 2.6. Preparation of (Bioactive) Chitosan Aerogels

Aerogel production started with the preparation of a chitosan (CH) stock solution at 2% (*w*/*w*) in 1% (*v*/*v*) aqueous acetic acid. This stock solution was mixed with distilled water until reaching 0.5% (*w*/*w*) solids content. Then, this solution was poured into 3 cm diameter plastic beakers. Subsequently, the samples were freeze-dried at −80 °C for 72 h with a vacuum of 0.5 Pa (LyoQuest, Telstar, Barcelona, Spain). After this procedure, blank aerogels (100% CH) were obtained. To construct CH aerogels reinforced with LCMNF or CMNF, 1% LCMNF and 0.5% CMNF aqueous slurry were used, respectively. The required amount of these suspensions (Table 1) was dispersed in distilled water in a mixer homogenizer (IKA T18 digital Ultra Turrax). Then, these suspensions were mixed with the required amount of CH stock solution followed by continuous stirring. The mixture was raised to pH 5.60 via 1 M NaOH and then glutaraldehyde (2% of CH powder) was added in the mixture and magnetically stirred for 24 h, as proposed by Zhang et al. (2021) [24]. Finally, these mixtures were poured into plastic beakers and freeze-dried as mentioned above. After this procedure, CH aerogels reinforced with LCMNF or CMNF were obtained and labeled as shown in Table 1.

Bioactive aerogels were prepared in the same way as described for the previously described aerogels, adding the BT together with the glutaraldehyde to attain final concentrations of 0.3, 0.7, 1, 2, 5, 10, and 20% (with regard to the total solids content). The rest of the procedure was followed as mentioned. The resulting bioactive aerogels were labeled as displayed in Table 1.

### 2.7. Characterization of BTPW Micro/Nanofiber-Reinforced CH Aerogels

Aerogels were characterized in terms of FTIR, XRD, SEM, density, porosity, mechanical performance, and water and oil absorption capacities. FTIR, XRD, and SEM analyses were performed as previously mentioned.

Density of the aerogels was determined from the dimension and weight of each aerogel. The obtained value was used to calculate the porosity as described by Geng, 2018 [38].

Mechanical performance tests were conducted on an Instron LF Plus Lloyd Instrument testing machine provided with a 1 kN load cell and a strain rate of 2 mm/min with a strain of 80%.

Water and oil absorption capacity of the prepared aerogels was determined as reported by Fontes-Candia et al. [1]. Thus, a square aerogel of 1 cm^2^ was weighed and immersed in a Falcon tube containing 15 mL of distilled water or soybean oil. After 24 h, samples were taken out and weighed after removing the liquid excess. These data were used to calculate the water and oil absorption capacity of the aerogels. The measurements were replicated 5 times and the results were expressed as an average with their relative standard deviation.

### 2.8. Characterization of Bioactive Aerogels

In addition to the same characterization carried out for the previous aerogels, the antioxidant activity and their use as food absorbent pads were tested.

#### 2.8.1. Radical Scavenging Activity by DPPH Assay

The antioxidant activity prolonged in time as a direct indicator of the release kinetics of BT in the bioactive aerogels was measured by DPPH assay [39]. Briefly, bioactive aerogel samples (10 mg) were weighed into different Falcon tubes with subsequent addition of 4 mL 0.2 mM DPPH methanolic solution. The tubes were allowed to stand out of the light, and after 30 min, the absorbance of the samples was measured at 517 nm. Additionally, at appropriate time intervals (until 48 h), the absorbance of the samples was recorded, as indicative of BT in vitro release. The ability to sequester the DPPH radical, expressed as percentage, was calculated in relation to the control (without antioxidant), according to the Equation (4).

(4) $$\%inhibition=\frac{ABS_{control}-{ABS}_{sample}}{ABS_{control}}\times 100$$

#### 2.8.2. Evaluation of the Effect of Bioactive Aerogels on Meat Preservation

Finally, the bioactive aerogels were tested as absorbent pads with burger meat and their preservation capacity was examined by the determination of oxymyoglobin and metmyoglobin content proportions in meat samples. Bioactive aerogels were placed covering the bottom surface of glass Petri dishes (Ø 55 mm) and portions of 2 g of minced burger red meat were placed on top of the aerogels. The samples were then sealed with parafilm and refrigerated at 4 °C for 10 days [1]. Control samples were prepared using commercial meat pads.

The ability of the bioactive aerogels to prevent meat oxidation was determined as a direct function of meat discoloration by measuring the proportion of oxymyoglobin and metmyoglobin forms as described by Fontes-Candia et al. with some modifications [1]. Thus, the meat samples were homogenized with 20 mL of 0.04 M potassium phosphate buffer (pH 6.8) and kept in the freezer for 1 h. Then, the samples were centrifuged at 4200 rpm for 30 min at 10 °C. The supernatant was filtered using 0.45 µm pore size filters and the volume of the sample was then adjusted with the buffer to 25 mL. Finally, the absorbance of the sample was measured at 503, 525, 557, and 582 nm in a spectrophotometer. The proportions of oxymyoglobin and metmyoglobin forms were calculated as proposed by Tang et al. [40] with the following equations:

(5) $$\%OxyMb=\left(0.722R_{1}-1.432R_{2}-1.659R_{3}+2.599\right)\times 100$$

(6) $$\%MetMb=\left(-0.159-0.085R_{2}+1.262R_{3}-0.520\right)\times 100$$

where *R*_1_, *R*_2_, and *R*_3_ are the absorbance ratios A^582^/A^525^, A^557^/A^525^, and A^503^/A^525^, respectively. All the determinations were carried out in triplicate. 

### 2.9. Statistics

All data have been represented as the average ± standard deviation. Analysis of variance (ANOVA) followed by the Duncan test were used when comparing water and soybean oil absorption capacities of aerogels. Different letters show significant differences (*p* ≤ 0.05).

## 3. Results and Discussion

### 3.1. Characterization of Bay Tree Pruning Waste Cellulose Fractions

BTPW was composed of 30.84 ± 0.39% cellulose, 17.58 ± 0.39% hemicelluloses, 22.31 ± 1.12% lignin, 4.87 ± 0.43% ash, and 17.05 ± 0.94% alcohol extractable [15]. After subjecting BTPW to a soda pulping process, optimized for maximum extraction yield using relatively mild procedures, unbleached cellulose pulp (UBP) was obtained. For this fraction, an increase from 48.42% to 72.23% in the carbohydrate fraction (sum of α-cellulose and hemicelluloses) compared to the raw material was determined, due to the purification of the cellulose fibers in the pulping process and a major part of hemicelluloses remaining [25]. After the bleaching process, the carbohydrate content was further increased (80.36%) due to the removal of a large proportion of the lignin in the bleached pulp (BP). The lignin content in UBP was 20.65 ± 1.13%, while after the bleaching step, it was 9.01 ± 0.21%. UBP showed a fiber length of 1514 nm, while BP was slightly shorter (1050 nm) with a degree of polymerization (DP) of 530 and 421, respectively, being attributed to the degradation of the fiber during the bleaching process. The BTPW cellulose fibers thus obtained resulted in similar characteristics to those reported for other agricultural residues such as olive pruning or tomato, bell pepper, and eggplant horticultural residues, in which a carbohydrate purification of almost 70% was achieved under the same pulping conditions [41,42].

The resulting BTPW-LCMNFs and CMNFs were characterized in terms of SEM (Figure S1), nanofibrillation yield, CD, and CC, and these data were used for the estimation of the specific surface area, diameter, polymerization degree, and length of the fibers. Several authors have previously reported these parameters as good indicators of the LCMNF and CMNF quality [43]. The nanofibrillation yield (η, Table 2) for LCMNFs was 48%, while for CMNFs, it was 58%. The lower performance in LCMNFs is associated with the presence of lignin, which causes interference during TEMPO-mediated oxidation, leading to secondary oxidation reactions [44].

CD represents the ability of the fibers to interact with their environment; therefore, a high CD value leads us to expect a higher electrostatic interaction between the nanofibers (anionic compound) and the chitosan (cationic compound). Regarding CD, there is a notable increase from LCMNFs to CMNFs (from 759 µeq/g to 1349 µeq/g). This lower CD in LCMNFs is directly related to the lower efficiency of the pretreatment on these fibers due to the high lignin content that decreases the amount of carboxyl groups introduced and a lower nanofibrillation, leading, in addition, to larger diameters in LCMNFs (Table 2). Similar values have been reported for LCMNFs from unbleached soda/oxygen sawdust pine pulp and CMNFs from commercial bleached kraft pine pulp. The same trend was observed for nanofibrillation yield of LCMNFs that was lower than CMNFs (55% and 62%, respectively) and for CD (899 and 1231 µeq/g, respectively) [45]. The DP of both LCMNFs and CMNFs was substantially decreased compared to the initial fiber, as was the length. It should be mentioned that the bleaching process affects the DP and therefore the length of the fibers, hence the decreased values in CMNFs. Thus, the characteristics of BTPW-LCMNFs and CMNFs were similar to those reported for other softwoods such as those obtained from dried softwood (e.g., spruce) [46].

Finally, the success of the procedures carried out was verified by analyzing the chemical structure of all the fractions by FTIR. The peak at 1600 cm^−1^ in LCMNFs and CMNFs assigned to the carboxyl groups after TEMPO-mediated oxidation, the peak at 1511 cm^−1^ related to the aromatic ring vibrations of lignin in the UBP and LCMNF spectra, and the peaks at 1026 cm^−1^ and 895 cm^−1^ indicative of cellulose purity were observed (Figure S2). The latter was corroborated by the XRD patterns (Figure S2), where the two peaks attributed to the diffraction planes of (1–10)/(110) and (200), which represent the typical cellulose I crystal form, were present [47].

### 3.2. Feasibility of BTPW-LCMNF and CMNF as an Enhancement for Chitosan Aerogels

After studying the characteristics of LCMNFs and CMNFs obtained from BTPW, they were used to enhance the properties of CH aerogels. Thus, to know the effect produced on the structure and to reach the optimal formulation, CH aerogels were formulated with increasing amounts of lignocellulose or cellulose micro/nanofibers (0, 1, 3, 5, 7, and 10%).

**Figure 1:**
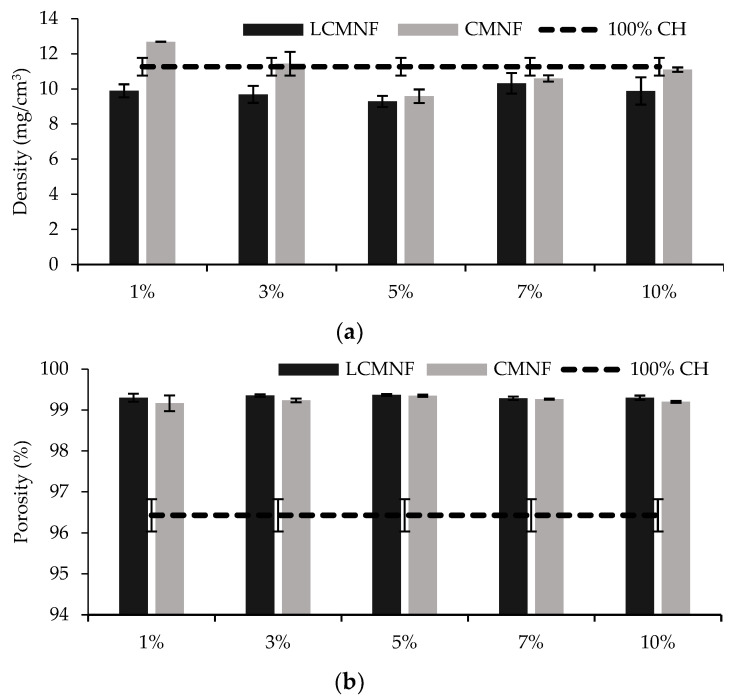
(**a**) Density and (**b**) porosity of the reinforced CH aerogels.

The density and porosity of the prepared aerogels is shown in Figure 1. In the case of LCMNF-CH aerogels, density slightly decreased when including BTPW-LCMNF at any concentration compared to 100% CH aerogel (from 11.26% to 9.28–9.68%). This can be explained by the crosslinking of the LCMNF-CH aerogels, which inhibits sliding of the building blocks and maintains the porous structure [24]. When CMNFs were used as the reinforcing agent, the aerogel density also slightly decreased compared to 100% CH, although this decrease was less pronounced than in the case of LCMNFs. Only the 1% CMNF aerogel slightly increased in density compared to the blank. Overall, the addition of micro/nanofibers reduced the structural defects in the aerogels, forming more porous and stable structures. The porosity of the aerogels increased in all cases when LCMNFs or CMNFs were added to the structure, due to the nanosize and high surface area of the nanocellulose [48]. As reported by Meng et al. (2017) for the fabrication of cellulose/chitosan composite aerogels, the low density and high porosity are beneficial characteristics to enhance the absorption capacities [49].

To find out the reaction mechanisms between the functional groups of LCMNF-CH and CMNF-CH aerogels, FTIR and XRD analyses were performed (Figure S3). However, no differences were observed between the FTIR spectra of the aerogels, finding the expected peaks for the presence of polymeric structures (CH and cellulose). Surprisingly, the effect of the crosslinker action was indeed determinant in the crystalline structure of the samples, as observed in the XRD patterns. Thus, when glutaraldehyde is not present, the diffractogram of the 100% CH aerogel corresponds to a typical polymeric amorphous structure. However, when the crosslinker agent is included, numerous peaks are observed, indicating in general the disappearance of the amorphous structures and the internal structure of the aerogel being ordered in some way to result in more crystalline materials. Regarding the FTIR spectra, some authors have suggested that the peak at 1550 cm^−1^ occurs as result of the appearance of amide bonds due to the crosslinking action between glutaraldehyde and CH [50]. However, this peak has been observed in 100% CH aerogels regardless of whether or not they were formulated with glutaraldehyde. Therefore, it is suggested that the interaction between CH and LCMNF/CMNF is by hydrogen bonds or electrostatic interaction since no detectable new functional groups appear. No new peaks were observed when the CH matrix was enhanced with LCMNFs or CMNFs. Therefore, these analyses indicated a correct crosslinking reaction between the different components of the aerogels without a modification of the basic chemical structure of the material upon addition of the BTPW fractions.

The morphology of the aerogels was examined using SEM, and representative images are given in Figure S4. The 100% CH aerogel exhibited an open pore network structure that was smoothed and homogenized when reinforced with both LCMNFs and CMNFs. No phase separation was observed, indicating good cohesion in the structure and successful crosslinking.

**Figure 2:**
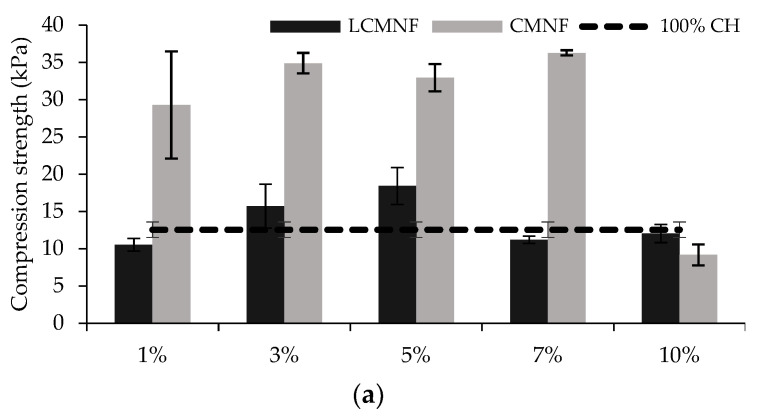

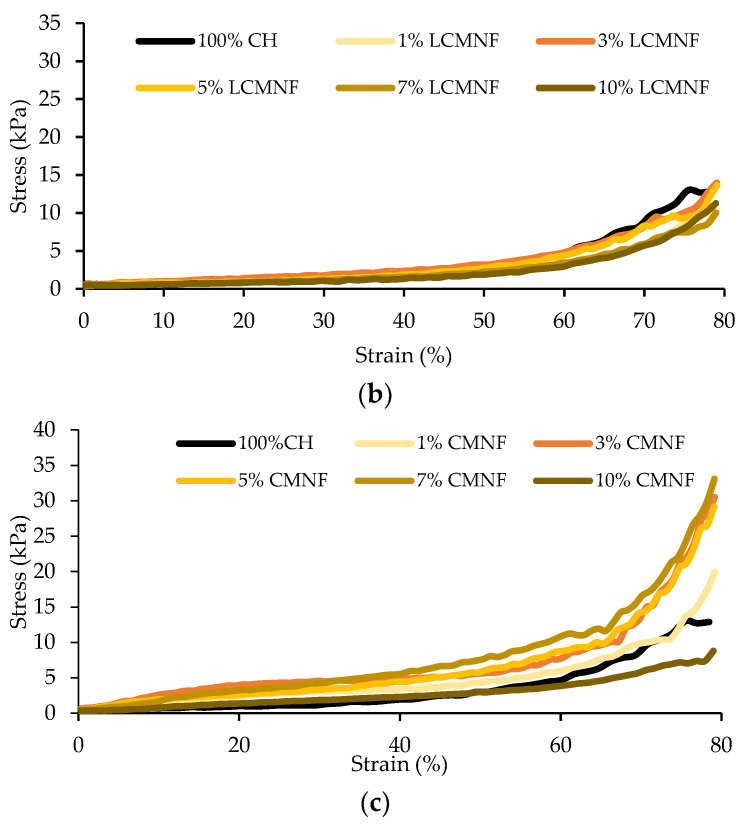
(**a**) Compressive strength, (**b**) stress-strain curves of the prepared LCMNF-CH and (**c**) CMNF-CH aerogels.

CH aerogels have weak mechanical performance, thus limiting their application. However, biomass-derived CNFs have proven to increase the mechanical strength in CH matrices [49]. In this study, it was observed how adding BTPW-LCMNFs strengthened the CH matrix starting at 3%, reaching the maximum reinforcing effect at 5% (18.41 kPa) compared to 100% CH (12.54 kPa). Higher LCMNF ratios (7–10%) did not imply a matrix reinforcing effect (Figure 2a). As previously mentioned, residual lignin in LCMNFs has been proven to be responsible for the high dispersion and reinforcing capacity in composite materials. Interestingly, the reinforcing effect was much greater when CMNFs were incorporated. Thus, CMNF aerogels increased their compressive strength with 3–7% CMNF (29.28–36.27 kPa). At 10% CMNF, this reinforcing effect is lost, possibly due to a collapse in the structure. The higher specific surface area achieved in CMNFs because it does not contain residual lignin compared to LCMNFs is responsible for this higher reinforcing capacity. As reported by Zhang et al. (2021) for anisotropic cellulose nanofiber/chitosan aerogels, nanofibers entangle into a 3D network more easily when lignin is not present, resulting in stronger adhesion and crosslinking between molecules [24]. In addition, the increase in Young’s modulus of CMNF-CH aerogels compared to 100% CH (Figure S5) suggests a higher elasticity of the materials, attributed to increased crosslinking, which is suitable for applications requiring flexible materials [51]. The strain–stress curves (Figure 2b,c) show how, at the beginning of the test, there is a slight increase in stress under compression due to the collapse of the porous structure forming a plateau region. When the strain increases above 40–50%, the stress begins to increase sharply due to the complete collapse of the cellular structures of the porous materials, forming a dense structure to resist deformation. This behavior is typical of foam-type materials [52,53,54]. In the case of CMNF aerogels, the maximum stress at 80% strain was 30–33 kPa for 3, 5, and 7% CMNF. Lower or higher concentrations of BTPW-CMNFs in the structure did not result in a reinforcing effect. The fact that the reinforcing effect is greater with CMNFs than with LCMNFs can again be attributed to the presence of lignin in LCMNFs. It has been recently reported that the presence of lignin interferes with the primary activator (NaClO) during the bleaching process, resulting in aerogels with poorer mechanical properties [44]. Some authors have reported that a higher aspect ratio of CMNFs results in a higher Young’s modulus of the aerogels when the content of crosslinker employed is less than that of micro/nanofibers [55]. BTPW-CMNFs exhibited a higher aspect ratio than BTPW-LCMNFs, thus confirming this explanation (Table 2).

**Figure 3:**
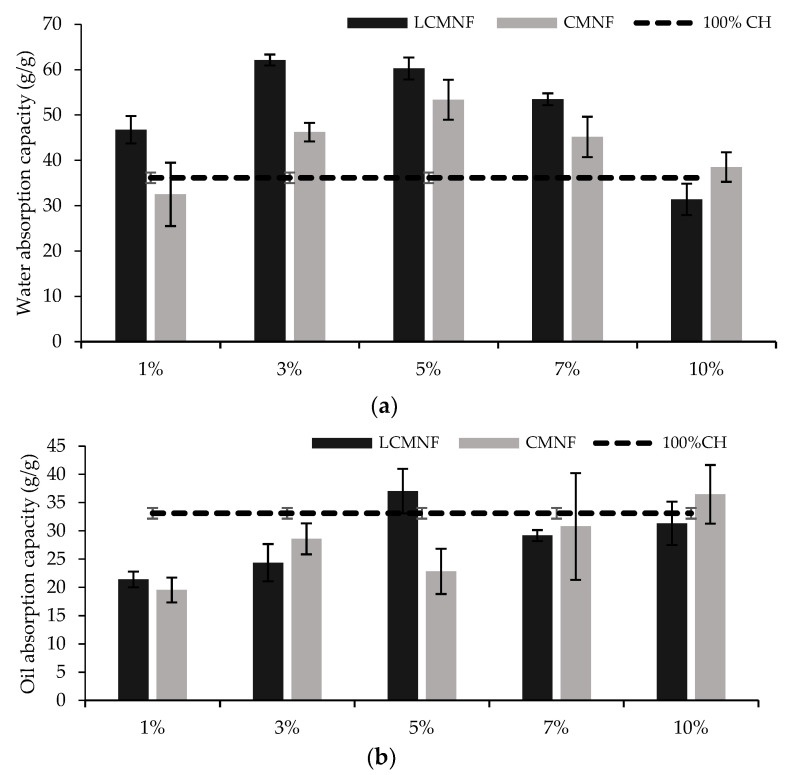
(**a**) Water and (**b**) soybean oil absorption capacity of LCMNF-CH and CMNF-CH aerogels. Bars with different letters are significantly different (*p* ≤ 0.05).

With the aerogels aimed to be applied in food packaging absorbent pads, a very porous structure with high absorption capacity is desired. The high porosity of aerogels containing LCMNFs and CMNFs has been previously proven. The results of the study of water and oil absorption capacity of aerogels are shown in Figure 3. In the case of water absorption, the incorporation of BTPW-LCMNFs into the CH matrix led to an increase in the absorption capacity. The maximum absorption capacities were reached with 3% LCMNF (62.12 g water/g aerogel) and 5% LCMNF (60.26 g water/g aerogel). When higher amounts were added, the water absorption capacity considerably decreased. Fontes-Candia et al. reported that high cellulose contents in the aerogel lead to the formation of stronger hydrogen bonding networks that decrease water uptake [1]. The values obtained in this study were slightly higher than those obtained for aerogels containing *Arundo donax* cellulose fractions and similar to those obtained for corn starch aerogels intended for active food packaging [1,18]. Only modified cellulose aerogels have higher water absorption capacities [24]. For CMNF aerogels, the maximum absorption capacity was reached at 5% (53.37% g water/g aerogel), being in all cases slightly lower than when LCMNFs were used. It is known that lignin is a hydrophobic fraction, and its presence in the fibers contributes to a lower hydrophilicity of the aerogels, thus decreasing water uptake [56]. However, in the case of BTPW-LCMNFs, it seems that the residual lignin contributes to a higher compatibility of the aerogels with water, since in the case of CMNFs, a more purified cellulose fraction, less hydroxyl groups would remain available in the aerogel pores due to a strong self-association [1]. In fact, the CD of CMNFs compared to LCMNFs (1349.02 µeq/g and 759.98 µeq/g, respectively) supports this theory, as previously mentioned in mechanical performance.

The oil absorption capacity of 100% CH was only slightly improved with 5% LCMNF (37.05 g oil/g aerogel compared to 33.12 g oil/g aerogel). In the case of LCMNF aerogels, oil absorption slightly increases with LCMNF content in low proportions (1–5%). This behavior could be attributed to a uniform alignment of numerous pores as a result of the increased dispersion due to the presence of residual lignin, which allows rapid liquid absorption and provides a large volume for storage of the absorbed liquids. When oil-absorbent materials are designed, absorption capacities reach 250 g/g [24]. When the aerogels were formulated with CMNFs, no significant differences were found in the concentration range 1–7%, the oil absorption capacity being 20–30 g oil/g aerogel. Therefore, the results obtained for the present aerogels suggest their suitability for absorption in hydrophilic matrices.

As mentioned above, when aerogels are designed to be food pads, they are required to be highly porous and absorbent. In the aerogels prepared so far, effective crosslinking between BTPW-LCMNFs or CMNFs and CH has been demonstrated, in addition to high porosity at all ratios studied (>99%). Moreover, a maximum reinforcing effect of the CH matrix was achieved with 5% LCMNF (23.41% improvement) and 3–7% CMNF (89.07% improvement), attributing these differences between LCMNFs and CMNFs to the higher aspect ratio of BTPW-CMNFs. Finally, the high water absorption capacity of the aerogels increased with 3–5% LCMNF and 5% CMNF. For oil absorption, the improvement over CH was achieved with 5% LCMNF. Considering these results, 5% LCMNF and 5% CMNF were selected as the optimum formulation of the CH aerogels, as a compromise between mechanical performance and absorption capacity.

### 3.3. Effect of Bay Leaf Extract on the Properties of Bioactive Aerogels

BT was obtained using the Soxhlet method and exhibited a TPC of 23.37 ± 4.09 GAE/g extract with 87% scavenging activity (SA) determined by the DPPH assay. These values were very similar and even higher than the ones reported for natural extracts aimed for food packaging applications, such as pinhão coat extract (22.53 GAE/g) or yerba-mate extract (12.88 GAE/g and 85% SA) [18,19].

**Figure 4:**
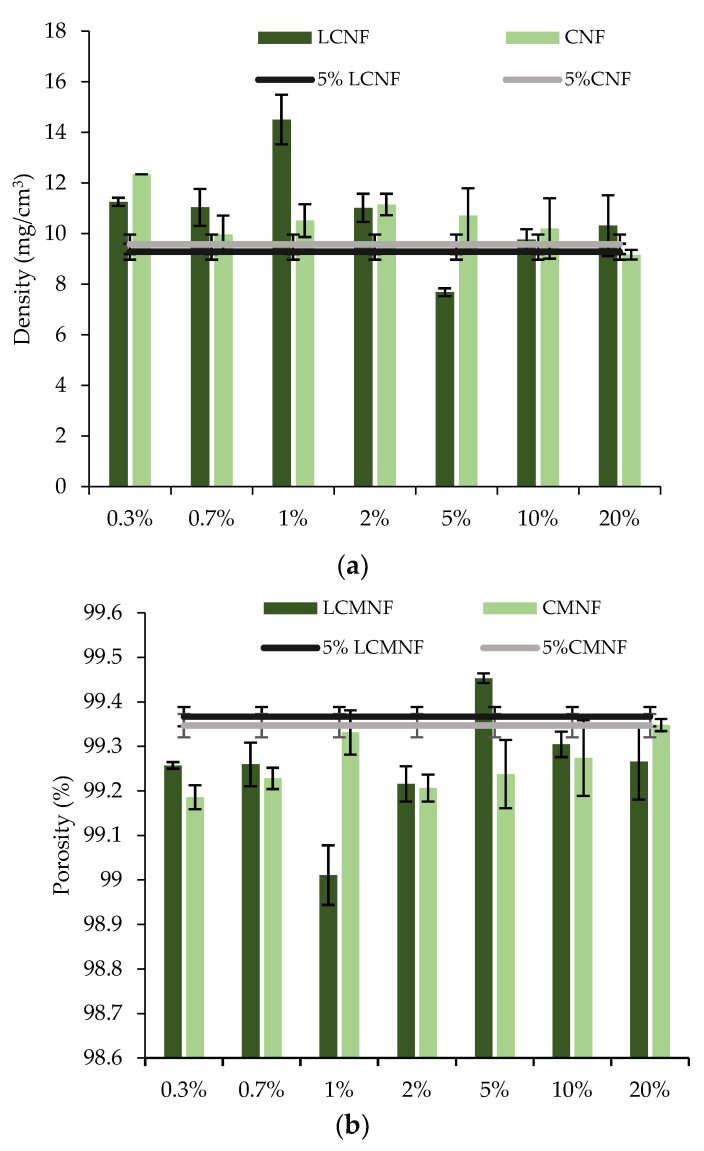
(**a**) Density and (**b**) porosity of bioactive aerogels.

Once the highly porous and absorbent aerogels as well as the BT extract had been obtained, we proceeded to formulate the bioactive aerogels by studying the effect of increasing concentrations of BT (0.3–20% BT) on 5% LCMNF and 5% CMNF aerogels. Density and porosity of bioactive aerogels are also shown in Figure 4. As expected, the bioactive aerogels slightly increased their density compared to their reference matrices because of the correct incorporation of BT. The porosity of the bioactive aerogels remained high (>99%) in all cases.

FTIR spectra of the bioactive aerogels (Figure S6) showed the same bands and peaks observed for the previous aerogels at low concentrations of BT (0.3–2%), indicating that no new functional groups are formed and that the retention of BT in the polymeric matrix could be purely a physical interaction. However, it is worth mentioning that, at high BT concentrations (5–20%), a new peak at 1730 cm^−1^ is observed in both LCMNF and CMNF aerogels, which is due to the presence of lipid compounds from the extract [3]. This peak probably does not appear in the spectra of low BT concentration bioactive aerogels because the amount incorporated is too low to be detected. Peaks at 1592 cm^−1^, 1412 cm^−1^, 1319 cm^−1^, and 1060 cm^−1^, typical of ethanolic BT, overlap with those of 5% LCMNF and 5% CMNF [31]. Similarities between all the spectra suggested incorporation of BT into the matrix of CH aerogels.

**Figure 5:**
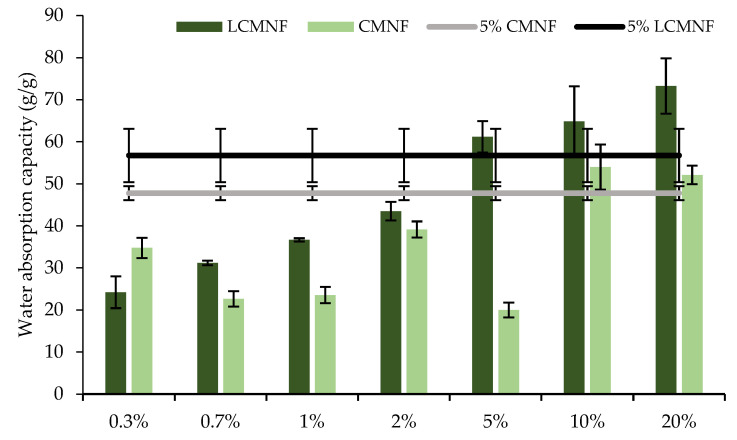

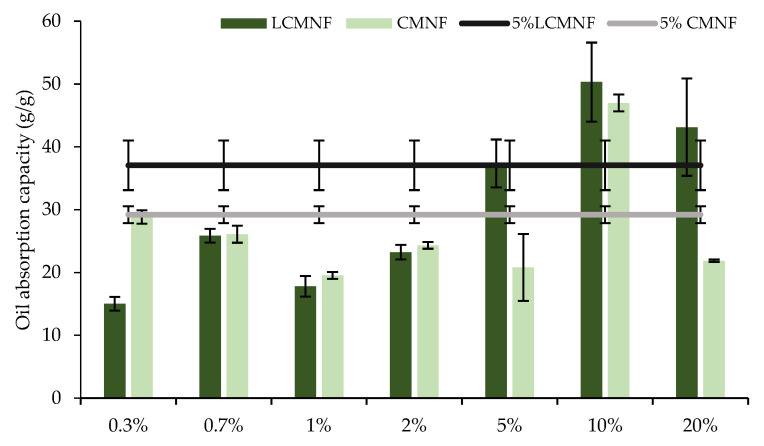
Water and soybean oil absorption capacities of bioactive LCMNF-CH and CMNF-CH aerogels.

The water and oil absorption capacities of the bioactive aerogels were again analyzed to verify they act as water or soybean oil absorbers after 24 h (Figure 5). Regarding the water uptake, the highest absorption capacities were obtained with concentrations ≥ 5% BT in the case of LCMNF-formulated aerogels. Thus, these aerogels exhibited a water uptake capacity of 61.17, 64.85, and 73.25 g water/g aerogel for 5, 10, and 20% BT, respectively, compared to 56.74 g water/g aerogel for 5% LCMNF. In the case of bioactive CMNF-formulated aerogels, a similar trend was observed, reaching the water uptake value of the reference with 10% BT. Kanmani and Rhim reported a slight increase in the water uptake ability of composite films when incorporating grapefruit seed extract, suggesting that it is due to the presence of groups with greater affinity to bind water molecules [57]. Similar behavior has also been reported for CH composites incorporating carvacrol and grapefruit seed extract [58]. As was the case with LCMNF-reinforced aerogels, the water uptake capacity was higher than with CMNF aerogels, corroborating the higher self-association of the fibers in the absence of residual lignin. This effect is even more remarkable when the aerogels are formulated with BT because, in the LCMNF aerogels, more BT will be retained, and therefore water will be retained both in the hydrophilic domains of BT and in the non-self-associated LCMNF due to the presence of lignin. In the case of soybean oil, absorption followed a similar trend, with maximum absorption values when the aerogels were formulated with 10% BT (50.29 and 46.29 g oil/g aerogel for LCMNF and CMNF, respectively). These values were higher than those reported for pure *Posidonia oceanica* (nano)cellulosic aerogels [59] or bio-aerogels from dried salad waste [60]. As in these cases, the absorption selectivity of the aerogels was higher for water than for oil. These facts make the bioactive aerogels prepared in this study applicable in food products with high aqueous activity or meats that release exudate, favoring its correct management [18].

Thus, the characterization carried out for the bioactive aerogels allowed us to verify the correct incorporation of BT in the aerogel matrix. The bioactive aerogels maintained the beneficial characteristics achieved with 5% LCMNF or 5% CMNF, such as high porosity and reinforced mechanical performance (Figure S7). At medium-high concentrations of BT in the aerogel matrix (5–20%), such important characteristics for the use of these materials as absorbent pads as water or oil absorption capacity were not only maintained with respect to their reference matrices but improved.

### 3.4. Evaluation of the Bioactive Capacity of BTPW-CH Aerogels for Meat Preservation

**Figure 6:**
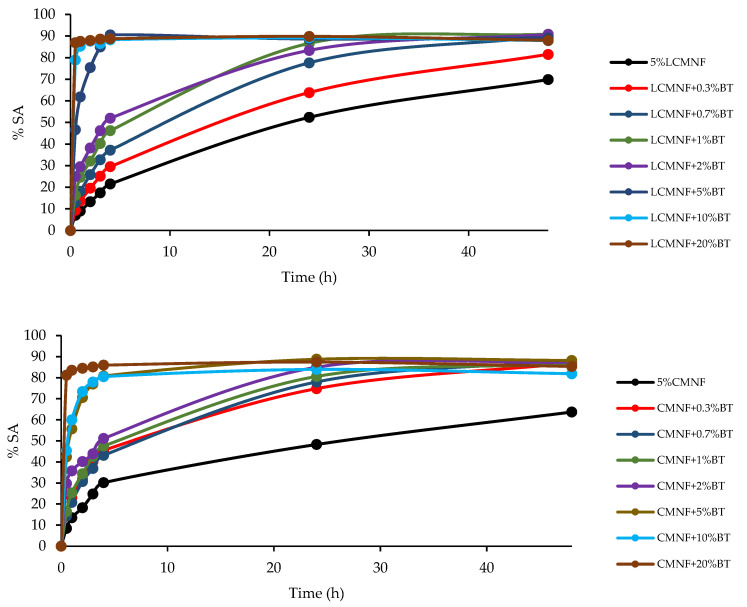
BT release profiles as function of DPPH scavenging activity (%SA) of bioactive LCMNF-CH and CMNF-CH aerogels.

The antioxidant capacity of the bioactive aerogels was evaluated as scavenging activity (%SA) by means of the DPPH assay prolonged in time, as a direct indicator of the release rate of the extract to the medium (Figure 6). For LCMNF aerogels, an increasing antioxidant power (%SA at 30 min) is observed as the concentration of BT in the aerogel increases. The 5% LCMNF aerogel exhibited 7.04, 52.38, and 69.86% SA for 0.5, 24, and 48 h, respectively, derived from the residual lignin content in LCMNFs, with an aromatic structure that acts as an antioxidant agent preventing or retarding oxidation processes induced by oxidizing species, such as free radicals [12]. Based on the behavior of the LCMNF aerogels in this test, they can be divided into two groups, those with ≤2% BT and those with ≥5% BT. Thus, the aerogels with low BT concentration in the matrix presented approximately 9, 13, 16, and 24% SA at 30 min of testing for 0.3, 0.7, 1, and 2% BT, respectively. During the first day of assay (0–24 h), it is shown how slowly the proportional release of BT to the medium (and therefore an increase of %SA) is taking place. After 48 h of testing, it could be said that aerogels containing 0.7–2% BT reached the maximum release and therefore scavenging activity (88–91% SA).

In the case of LCMNF aerogels containing high proportions of BT (5–20% BT), certain differences are observed in this behavior. After only 30 min of testing, 46.60, 78.92, and 86.95% SA were obtained for 5, 10, and 20% BT, respectively. For these aerogels, the BT release rate was much faster than in the previous ones. The 5% BT aerogel exhibited 61.86, 75.39, and 85.04% SA for 1, 2, and 3 h of assay, while from 4 h and up to 48 h, the %SA was at maximum. For 10% BT and 20% BT aerogels, the %SA was at maximum from 1 h of assay, indicating the even faster rate of BT release. These differences between aerogels with low and high BT ratios can be attributed to the fact that, at low ratios, there is less availability of extract to interact with DPPH because it is interacting with LCMNF in the aerogel matrix, while at high BT ratios, all the free hydroxyl groups of LCMNFs for interaction with BT would have already been filled, leaving BT still free to scavenge the DPPH radical which translates into higher %SA at shorter test times [19].

Bioactive CMNF aerogels showed a very similar behavior pattern to LCMNF aerogels with some slight differences. Thus, it is observed that, in the case of aerogels with high proportions of BT, the total release of the extract in the first hours of the test is done at a slightly lower rate than in LCMNF aerogels. For example, 10% BT CMNF exhibited 60.04 and 73.43% SA for 1 and 2 h trial, respectively, while 10% BT LCMNF exhibited 85.26 and 87.62% SA for 1 and 2 h trial, respectively. However, it is seen how at 24 h of assay, the %SA and, therefore, the release of the extract, is practically maximal for any proportion of BT in the CMNF aerogel matrix (0.3–20% BT). According to the results reported by Fontes-Candia et al., this behavior is explained by several facts. First, in the early stages of the assay, the release is more prominent for aerogels with a lower purified cellulose fraction (LCMNFs) due to the morphology of these aerogels. The results obtained for the absorption capacity of these samples were also higher than for CMNF aerogels because of the presence of residual lignin. This contributed to the faster release of the extract, which resulted in an increase in %SA at earlier stages. On the other hand, results for the bioactive CMNF aerogels had suggested a stronger BT–CMNF interaction, resulting in a slower release of the extract [1]. In addition to this, it is known that the specific surface area is one of the most important parameters controlling the dissolution rate of compounds and their absorption. Composite materials with high surface areas result in a slower and more gradual release profile, which is very interesting for food preservation and active packaging [19,61]. Thus, BTPW-CMNFs presented a higher specific surface than BTPW-LCMNFs (Table 2), supporting the release behavior found. In any case, the BT extract presents a polyphenol-rich profile with high antioxidant capacity [29], being possible to suggest that polymeric matrices loaded with BT, such as the ones that compose the present aerogels, present a great potential for the formulation of food preservatives and nutraceuticals.

**Figure 7:**
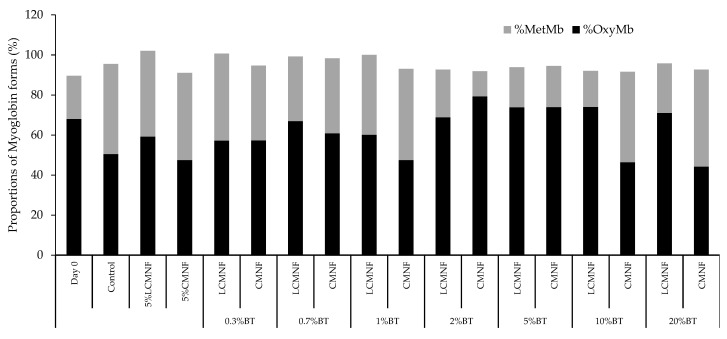
Proportions of oxymyoglobin (%OxyMb) and metmyoglobin (%MetMb) in the raw minced red meat (day 0) and in the meat after 10 days of storage.

As a final proof of concept, the prepared bioactive aerogels were tested as absorbent pads to inhibit the color loss of burger minced meat, directly related to lipid oxidation and thus rancidity. Meat discoloration is the consequence of the formation of metmyoglobin species as result of the oxymyoglobin oxidation process. The intensity in the meat color is due to the total myoglobin content. The stability of the color, therefore, is determined by the maintenance of myoglobin in its oxygenated ferrous form (oxymyoglobin). Oxidation of this form to ferric myoglobin (metmyoglobin) is the process that takes place during storage of refrigerated meat and is responsible for rancidity [62]. The proportion of these two forms in the meat was estimated after storage for 10 days in contact with the absorbent pads and the results are shown in Figure 7. On day 0, as expected with fresh meat, the content of oxymyoglobin was higher than that of metmyoglobin. After 10 days of refrigerated storage (control sample), the proportion of these two species varied, with the proportion of metmyoglobin increasing, indicative of color loss.

Interestingly, when the burger meat was stored with the bioactive pads, the appearance of metmyoglobin in the sample after 10 days of storage was lower than in the control sample, reaching in the best cases a metmyoglobin proportion of 20% compared to 45% for the control pad. Similar behavior has been reported for *Arundo donax* cellulose-based aerogels [1], which obtained 46% metmyoglobin forms compared to 65% in the control pad. In the case of this study, when burger meat was stored with the LCMNF pads, there was a slight increase in the proportion of oxymyoglobin for 5% LCMNF, 0.3% BT, 0.7% BT, and 1% BT, compared to the control sample. The most important fact was that for BT concentrations ≥2%, the proportion of oxymyoglobin in the meat was the same as that obtained in the fresh meat on day 0 of the trial, highlighting the great potential of these materials to limit oxidation processes in meat foods. When the meat was refrigerated with the CMNF pads, the same trend was observed with the difference that the 5% CMNF pad did not lead to an increase in the proportion of oxymyoglobin with respect to the control sample, as was the case with 5% LCMNF. This indicates that the presence of residual lignin in LCMNFs is contributing to this food maintenance, as suggested by the %SA result for this aerogel. Finally, differences were observed between pads reinforced with LCMNFs or CMNFs containing high concentrations of the extract (10–20% BT). Thus, for LCMNF pads, the results obtained were the same as those mentioned above for BT concentrations ≥2% BT; the proportion of oxymyoglobin remained similar to that of the fresh meat on day 0. However, for CMNF pads, the proportion of oxymyoglobin was somehow in line with that of the samples containing low concentrations of BT (0.3–1% BT). Maybe, for these CMNF-pads, the bioactive fraction has been released to the medium too fast, hindering on the one hand the correct absorption of the meat exudate and on the other hand not carrying out its antioxidative effect.

## 4. Conclusions

The present study has addressed the preparation of absorbent food pads in the context of agricultural residues biorefinery. For this purpose, bay tree pruning waste (BTPW) has been used for the production of lignocellulose and cellulose micro/nanofibers (LCMNF and CMNF). CMNFs were obtained with higher cationic demand and aspect ratio (1349 µeq/g and 132.90, respectively) compared to LCMNFs (759 µeq/g and 96.65, respectively). These are key factors when applying these fractions in food pads, as they are directly related to the mechanical performance and absorption properties. LCMNF and CMNF were applied as reinforcing agents in chitosan aerogels in order to elucidate the optimal formulation to be applied as food pads, together with the evaluation of the presence of residual lignin. Although the use of LCMNFs resulted in an optimal reinforcing effect at 5%, it was considerably greater (80% improvement) when using CMNFs. The absence of residual lignin in the latter seems to have contributed to a stronger self-association between the fibers, increasing the compressive strength. On the other hand, the presence of residual lignin in LCMNF aerogels was decisive in increasing the water absorption capacity of the materials, which reached their maximum with 3–5% LCMNF (62.12 and 60.26 g water/g aerogel). The 5% LCMNF and 5% CMNF aerogels were selected as the optimal formulation. These aerogels were prepared with bay leaf extracts (BT) to make them bioactive. The study of the antioxidant capacity of the aerogels prolonged in time, as a direct indicator of the release rate of BT to the medium, proved that the presence of residual lignin led to a faster increase in antioxidant power in the first hours of the assay (80% antioxidant power in 30 min). In both cases, aerogels showed great potential for the formulation of active food packaging materials. Finally, the use of these bioactive aerogels as absorbent pads for burger meat resulted in a maintenance of the freshness of the meat product after 10 days of storage. Again, the residual lignin present was a key factor since, in the case of 5% LCMNF pads, this retardation of meat oxidation was obtained with lower amounts of BT in the formulation as a result of a synergistic effect between the residual lignin and the extract.

## Figures and Tables

**Table 1:** Formulated (bioactive) aerogels composition.

Aerogel Sample	CH (%)	LCMNF (%)	CMNF (%)	BT (%)
100% CH	100	-	-	-
1% LCMNF	99	1		
3% LCMNF	97	3		
5% LCMNF	95	5		
7% LCMNF	93	7		
10% LCMNF	90	10		
1% CMNF	99	-	1	-
3% CMNF	97		3	
5% CMNF	95		5	
7% CMNF	93		7	
10% CMNF	90		10	
Bioactive aerogel sample				
LCMNF + 0.3% BT	95	5	-	0.3
LCMNF + 0.7% BT				0.7
LCMNF + 1% BT				1
LCMNF + 2% BT				2
LCMNF + 5% BT				5
LCMNF + 10% BT				10
LCMNF + 20% BT				20
CMNF + 0.3% BT	95	-	5	0.3
CMNF + 0.7% BT				0.7
CMNF + 1% BT				1
CMNF + 2% BT				2
CMNF + 5% BT				5
CMNF + 10% BT				10
CMNF + 20% BT				20

**Table 2:** Characterization of LCMNFs and CMNFs from bay tree pruning waste (BTPW).

	η (%) ^a^	CD (µeq/g) ^b^	CC (µeq /g) ^c^	Specific Surface (m^2^/g)	D (nm) ^d^	Length (nm)	Aspect Ratio
LCMNF	48.06 ± 5.04	759.98 ± 38.38	146.11 ± 32.72	300	8.33	805.12	96.65
CMNF	58.763 ± 11.76	1349.02 ± 3.29	141.18 ± 23.57	591	4.23	562.17	132.90

^a^ η—nanofribillation yield (%); ^b^ CD—cationic demand (µeq/g); ^c^ CC—carboxyl content (meq/g); ^d^ D—diameter (nm).

## Data Availability

The dataset generated and/or analyzed during the current study are available from the corresponding author on reasonable request.

## Supplementary Materials

The following supporting information can be downloaded at: https://www.mdpi.com/article/10.3390/polym15040866/s1, Figure S1: SEM micrographs of (a) unbleached cellulose pulp, (b) bleached cellulose pulp and (c) nanofibrillation process of the fiber; Figure S2: FTIR spectra (a) and XRD patterns (b) of bay tree pruning unbleached and bleached pulp, lignocellulose micro/nanofibers (LCMNF) and cellulose micro/nanofibers (CMNF); Figure S3: FTIR spectra (a,b) and XRD patterns (c,d) of 100% CH, LCMNF-CH and CMNF-CH aerogels; Figure S4: SEM images of (a) 100% CH, (b) 5% LCMNF, (c) 5% CMNF, and (d) LCMNF + 20% BT aerogels; Figure S5: Young’s Modulus of reinforced-CH aerogels; Figure S6: FTIR spectra of bioactive aerogels; Figure S7: Mechanical properties of bioactive aerogels.
