# Supplementary material for: Bioactive Absorbent Chitosan Aerogels Reinforced with Bay Tree Pruning Waste Nanocellulose with Antioxidant Properties for Burger Meat Preservation
Source: Polymers (Basel). 2023 Feb 9;15(4):866. doi: 10.3390/polym15040866 (PMC9964863; doi:10.3390/polym15040866)

polymers

Supplementary Materials

Bioactive absorbent chitosan aerogels reinforced with bay tree pruning waste nanocellulose with antioxidant properties for burger meat preservation

Figure S1. SEM micrographs of (a) unbleached cellulose pulp, (b) bleached cellulose pulp and (c) nanofibrillation process of the fiber.

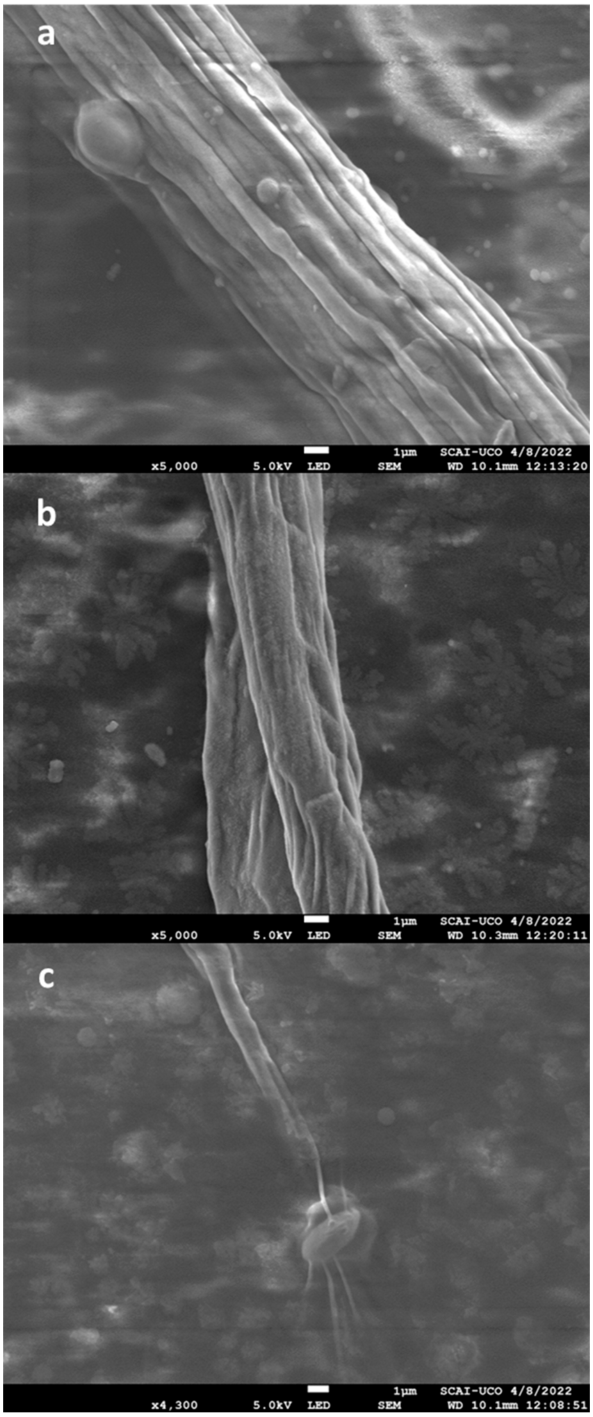

Figure S2. FTIR spectra (a) and XRD patterns (b) of bay tree pruning unbleached and bleached pulp, lignocellulose micro/nanofibers (LCMNF) and cellulose micro/nanofibers (CMNF).

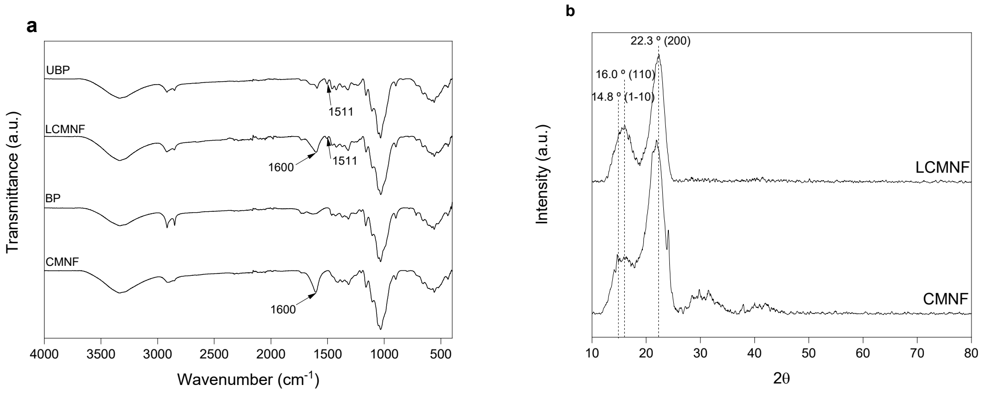

Figure S3. FTIR spectra (a,b) and XRD patterns (c,d) of 100% CH, LCMNF-CH and CMNF-CH aerogels.

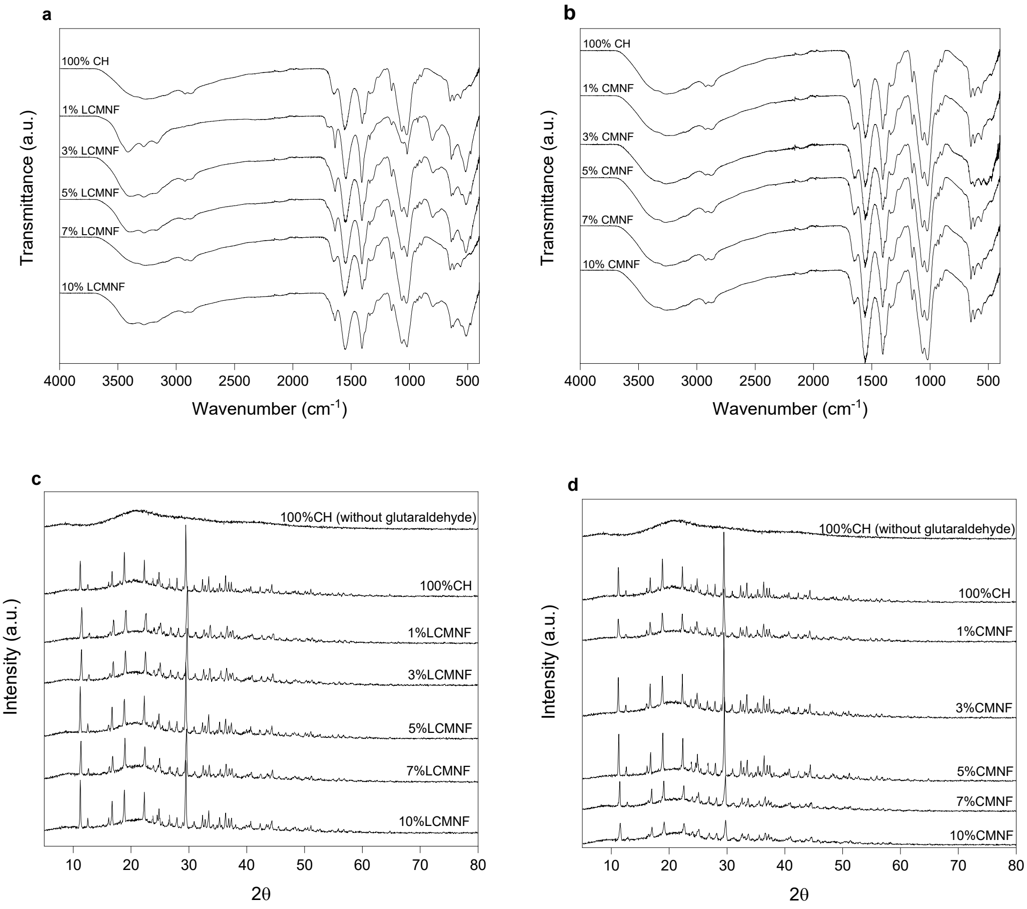

Figure S4. SEM images of (a) 100%CH, (b)5%LCMNF, (c)5%CMNF and (d)LCMNF+20%BT aerogels.

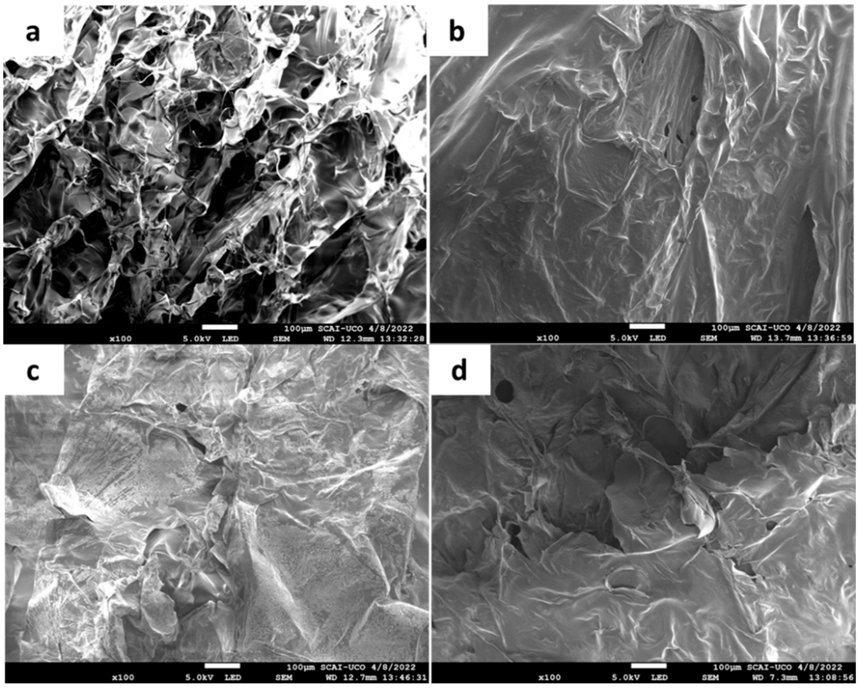

Figure S5. Young's Modulus of reinforced-CH aerogels.

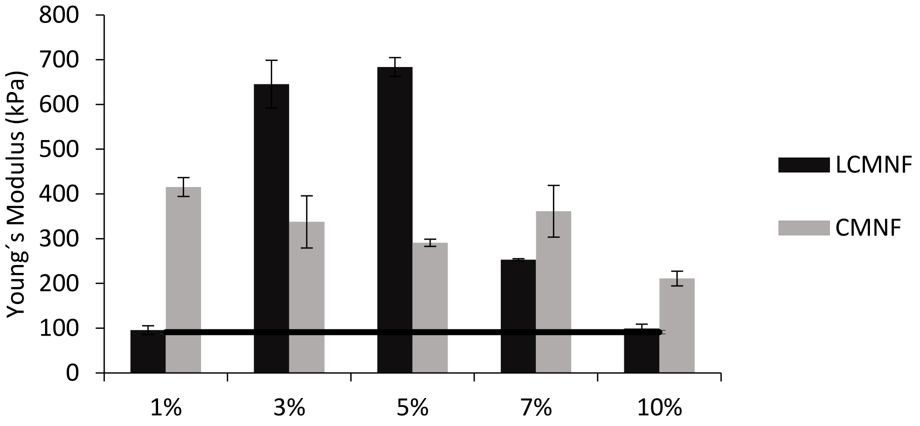

Figure S6. FTIR spectra of bioactive aerogels.

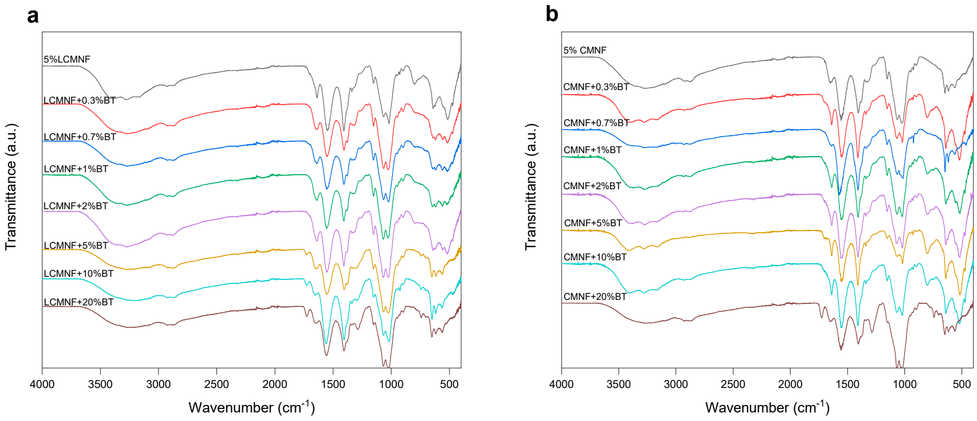

Figure S7. Mechanical properties of bioactive aerogels.

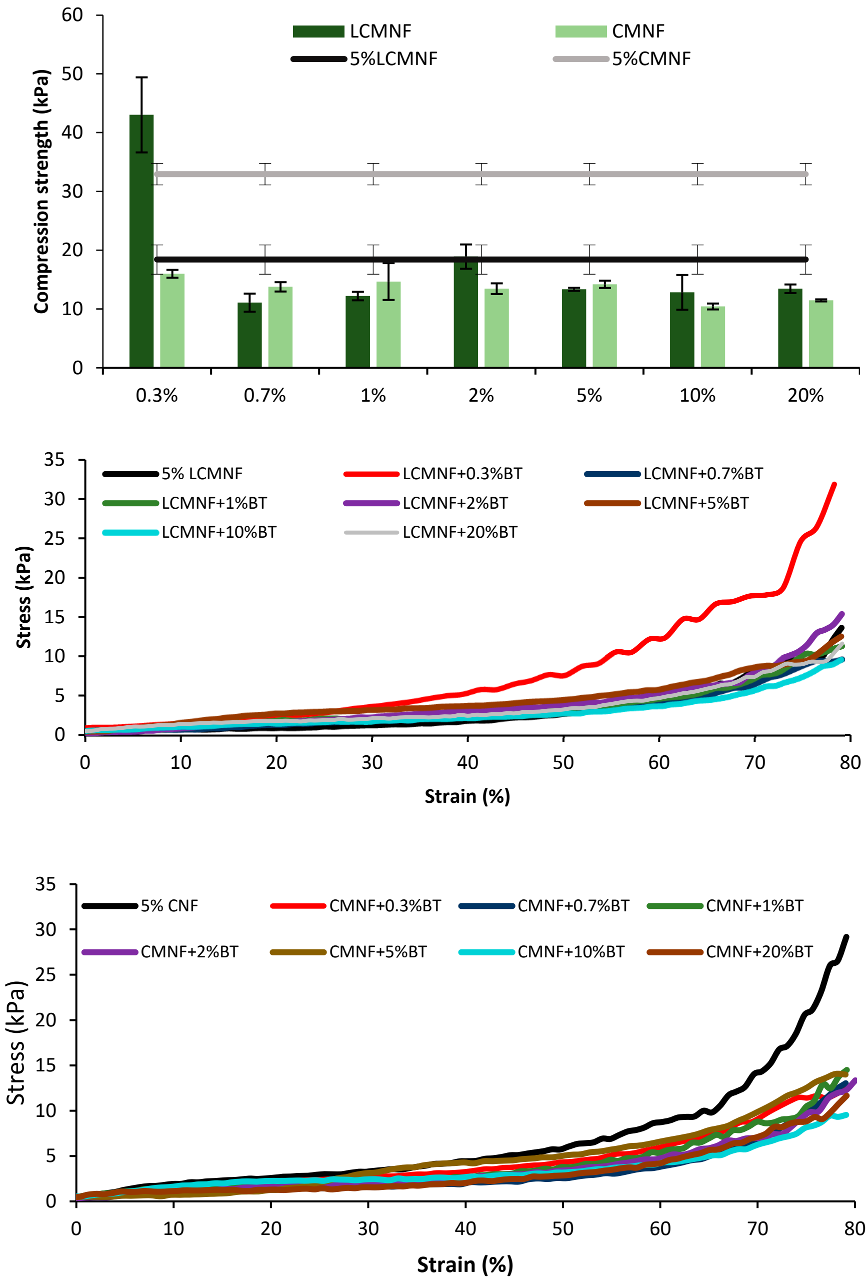

Supplement: Supplementary file 1 [file polymers-15-00866-s001.zip › polymers-2183714-supplementary.pdf]
